# Impact of Covid-19 on the urology service in United States: perspectives and strategies to face a Pandemic

**DOI:** 10.1590/S1677-5538.IBJU.2020.S126

**Published:** 2020-07-27

**Authors:** Giovanni Enrico Cacciamani, Mihir Shah, Wesley Yip, Andre Abreu, Daniel Park, Gerhard Fuchs

**Affiliations:** 1 USC/Norris Comprehensive Cancer Center Keck School of Medicine USC Institute of Urology Los AngelesCA US Department of Urology, Catherine & Joseph Aresty, USC Institute of Urology, Keck School of Medicine, USC/Norris Comprehensive Cancer Center, Los Angeles, CA, US; 2 Univ. of Southern California Department of Radiology Los AngelesCA US Department of Radiology, Univ. of Southern California, Los Angeles, CA, US

**Keywords:** COVID-19 diagnostic testing [Supplementary Concept], Telemedicine, Surgical Procedures, Operative, Urology

## Abstract

Over the course of several weeks following the first diagnosed case of COVID-19 In the U.S., the virus rapidly spread across our communities. It became evident that the pandemic was going to place a severe strain on all components of the U.S. healthcare system, and we needed to adapt our daily practices, training and education. In the present paper we discuss four pillars to face a pandemic: surgical and outpatients service, tele-medicine and tele-education. In the face of unprecedented risks in providing adequate health care to our patients during this current, evolving public health crisis of COVID-19, alternative patient management tools such as telemedicine services, allow clinicians to maintain necessary patient rapport with their healthcare provider when required. As a subspecialty, urology should take full advantage of telehealth and teleeducation at this juncture. As tele-urology and tele-education can obviate the potential drawbacks of “social distancing” as it pertains to healthcare, the platform can also reduce the risk of COVID-19 spread, without compromising quality urological care and educational efforts. Telehealth can bring urologists and their patients together, perhaps closer than ever.

## INTRODUCTION

The novel coronavirus known as severe acute respiratory syndrome corona virus-2 (SARS-CoV-2) has rapidly spread across the globe causing a pandemic known as the Coronavirus Disease 2019 (COVID-19) (1). On March 11, 2020, the World Health Organization (WHO) defined COVID-19 as a pandemic, and two days later, the President of the United States (U.S.) declared the COVID-19 outbreak a national emergency.

Since the first reported case in the U.S. (2), the healthcare community had been bracing for possible community spread of COVID-19 and its potential impact on the U.S. healthcare system. The U.S. was warned of what to expect by our healthcare colleagues across the globe, especially in Italy, where the virus' impact had been felt several weeks earlier than the U.S. (3). As in Asia and in some European countries, widespread transmission of COVID-19 in the U.S. was likely to occur with the majority of the U.S. population becoming exposed to the virus and potentially contracting COVID-19.

Over the course of several weeks following the first diagnosed case of COVID-19 in the U.S., the virus rapidly spread across our communities. It became evident that the pandemic was going to place a severe strain on all components of the U.S. healthcare system, and we needed to adapt our practices quickly to allow us to care for a surging number of COVID-19 positive patients. According to the Centers for Disease Control and Prevention (CDC), more cases of COVID-19 are likely to be identified in the U.S. in the next weeks, requiring stricter measures to reduce community spread. Moreover, the pandemic is placing a heavy demand on resources such as personal protective equipment (PPE), intensive care unit (ICU) beds, ventilators, medical supplies, as well as appropriately trained health care professionals. It became obvious that healthcare systems needed to reorganize to avoid being overwhelmed.

### Impact of COVID-19 on the surgical service

On March 18th, 2020 the Centers for Medicare εt Medicaid Services (CMS), a federal agency within the U.S. Department of Health and Human Services, formally released recommendations to delay all elective surgeries and non-essential procedures. In response, professional bodies began to make recommendations to help in re-prioritizing surgical cases and healthcare systems began to create an individualized plan for their institution to prepare for a surge in COVID-19 cases and how they would meet the challenges (4).

Urologists across the U.S. began deferring elective procedures and triaged their surgical cases (5, 6). Publications addressing management of genitourinary cancer care as well as kidney stone patients during the COVID-19 pandemic have helped guide our care of the urologic patient (3, 7). It is important to delay non-urgent cases not only to conserve medical resources but also to protect our patients as well as healthcare workers from potentially being exposed to COVID-19. In the worst hit regions of the U.S. such as New York, urologists were redeployed to assist with care of COVID-19 patients (8, 9). But even for those who are not redeployed to the emergency room and ICU, there are several additional preoperative considerations when operating during the COVID-19 era. The surgeon must weigh the risks to the patient from their underlying disease necessitating surgery versus the risk from possible COVID-19 exposure. A significant population of urologic patients are older with multiple comorbidities, placing them at a greater risk of worse outcomes if they were to contract the novel coronavirus (10). Those patients with genitourinary malignancies face even greater risk than their age matched cohort, as cancer patients are noted to have higher risk and more severe outcomes in a study from China (11). There is a national shortage of blood products during this pandemic and one must be judicious with transfusing their patients as well as weight the need for blood products during the perioperative period when considering proceeding with a surgery (12). As studies have indicated, a significant portion, up to 60% of patients whom are infected with COVID-19, may display minimal to no symptoms, yet be contagious and further spread the virus (13). It is prudent to screen every patient scheduled for a surgical procedure with not only with a comprehensive history and physical exam, but also with COVID-19 screening 1-3 days preoperatively to identify potential asymptomatic carriers and consequently delay their procedure as these patients are shown to have a 20% mortality rate if undiagnosed and undergo surgery during the incubation period (14).

Patients that need urgent surgeries are brought to the operating room (OR) with stringent rules and restrictions in place to mitigate the spread of COVID-19. Specific PPE guidelines have been adopted across U.S. hospitals to appropriately resource the insufficiently available masks and gowns while protecting their clinicians and staff. Aerosolizing procedures such as intubation require N95 masks with face shields for all non-COVID positive patients, while a Powered Air-Purifying Respirator (PAPR) is required for all COVID-19 positive patients (15). All staff except for the anesthesia team are asked to step outside the OR for intubation as well as extubation to minimize exposure risk. In addition, for those patients whom are suspect or have tested positive, staff should remain outside the room for at least 18 minutes to remove 99% of aerosolized virus in a negative pressure room (assumes ACH 15/hr) (16). Urologists must be aware that the virus is shed not only during aerosolizing procedures, but can also be shed in blood, urine and feces (17). During laparoscopic and robotic procedures, there is a theoretical risk of aerosolizing the virus therefore caution must be taken to suction gases into a closed system during de-sufflation of pneumo-peritoneum, and the OR staff must wear N95 masks throughout the case to limit possible exposure (18). For those performing robotic surgeries, surgeons should consider donning masks and gloves at the surgical console to minimize the exposure to COVID-19. It is critical for teaching institutions to protect their trainees and limit exposure during this COVID-19 pandemic. Academic institutions have modified and restructured their training programs to minimize exposure to their residents and fellows, as well as avoid any non-essential personnel such as visiting urologists, medical students, and researchers in the OR (19).

Post-operative care of our patients is also different during the COVID-19 era as entire hospital wards have been transformed to care for COVID patients. Urology patients are placed on non-COVID floors, but in some cases, this can lead to ancillary care provided by nurses not familiar with the management of a post-operative urology patient. Hospitals across the U.S. have adopted stern policies limiting any patient visitors per CDC recommendations and this has led to our surgical patients unable to have visitors during their hospital stay (20). Further, the patient care team must carefully weigh the need for post-hospitalization rehabilitation for our patients as placement to nursing homes and long-term care facilities can subsequently place them at a higher risk of contracting COVID-19. Additionally, it is critical to inquire about our patients after discharge regularly either via phone call or telemedicine to confirm an uneventful recovery while convalescing at home to help minimize possible readmissions.

### Impact of COVID-19 on the outpatient service

No facet of the U.S. healthcare system has been spared by the COVID-19 pandemic and outpatient services are no different. As we continue to make every effort to mitigate the spread of the virus, it is important to maintain social distancing, even within the hospital and outpatient clinics. As healthcare systems began to restructure and organize their resources and personnel to prepare for a surge in COVID-19 patients, outpatient visits were reduced to only those deemed absolutely necessary while all others were switched over to the rapidly adopted telemedicine platform. On March 17, 2020 CMS announced it had lifted restrictions on billing for telemedicine visits facilitating the wide adoption of telehealth during the pandemic (21). Minimizing traffic at the outpatient clinics allowed for appropriate social distancing, medical resource conservation, and limiting exposure risks to patients and staff alike. Based on CDC guidelines, patients checking in for in-person visits were screened for any symptoms of COVID-19 over the phone at the time of scheduling their appointment as well as upon arrival to the facility and are provided with a mask at check-in to mitigate the spread of the infection (22). All front and back office staff should wear masks and PPE as indicated by CDC guidelines to minimize any exposure. While most office visits were changed to telemedicine visits, there are patients still requiring clinic procedures and in-person visits. Recommendations for triaging office procedures have been made by Katz et. al as well as Howard et. al. to help guide the efforts to limit any non-urgent procedures (5, 23). While this transition away from in-person clinic visits has presented new challenges for the provider and the patient, the U.S. healthcare community has risen to meet these demands with some changes likely to stay in place beyond the COVID era.

### Impact of COVID-19 on Telemedicine

Following the concept and step-wise restrictions of *#Stayathome* mandates, millions of Americans have had to restrict their daily activities, avoiding public areas, public transportation, and reduce physical contact to limit the risk of person-to-person transmission. Under these circumstances, telehealth represents the venue for reaching these goals without limiting access to healthcare or compromising patients' health unduly. The concept of the tele-visit employs telecommunication tools to share healthcare information between patients and providers. Several communication tools have been described for two-way audio-video platforms such as computers, touchpads, and smartphones (24).

According to a 2019 survey, only 10% of Americans have used telehealth for a virtual consultation with lack of access (34.6%) and poor awareness (39.7%) of telehealth options as the primary hurdles to adoption (25). The COVID-19 outbreaks and the restrictions suggested by the CDC with stepwise implementation by government agencies for containing the spread of contagion would ultimately bring telehealth into the mainstream of practices, thereby reshaping the future of access to healthcare. In this setting, tele-urology could provide an alternative setting to evaluate post-operative patients. The tele-visit would make it easier for patients and providers to connect, while reducing person-to-person contact with public transportation, in various waiting rooms, hospitals, and clinics, including the urologist and their staff.

Patient acceptance and perceptions of telehealth for new patient visits, follow-up visits, clinic, and hospital consultations have been previously explored, showing potential for improving the urologic continuum of care. Younger patients (mean 62 vs. 65 years), higher education level (77% vs. 65%), previous exposure to video-conference tools (57% vs. 38%), those travelling longer distances (>90 min; 69% vs. 58%), and days missed from work (>1 day; 39% vs. 29%), have been found to prefer the tele-visit setting for sharing new symptoms and sensitive information (26). Viers et al. reported the use of tele-visits for patients following prostatectomy. No significant differences were found in patient perception of the quality of care nor satisfaction with the visit, with similar patient-to-provider face time (14.5 min vs. 14.3 min), patient wait time (18.4 min vs. 13.0 min), and total time devoted to care (17.9 min vs. 17.8 min). Likewise, there were no differences with the urologists' perspective. Further, overall costs to patients have been found to be lower with the tele-visit (27).

**Figure 1:**
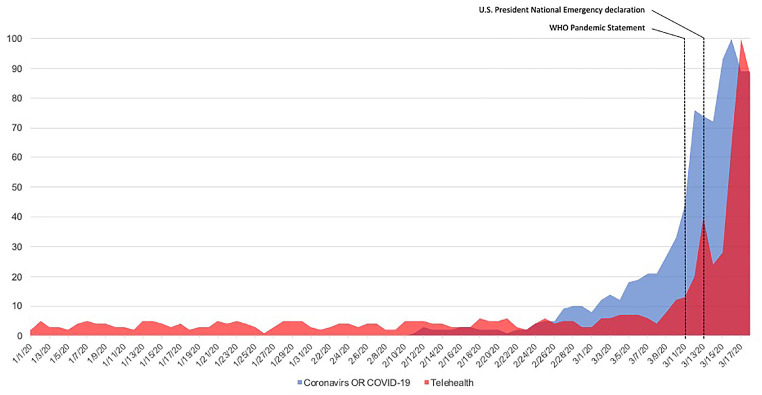
Google trends analysis for “Tele-Health OR Telehealth” and Corona Virus. Data are reported in relative search volume.

While tele-urology showed encouraging results, up until now it had only been offered as an option. In our current pandemic setting with increasing spread of the COVID virus and mobility restrictions, it might be necessary to employ telehealth to maintain patient access to healthcare. As regulatory barriers for the use of telehealth systems have been tabled for now, urologists should take the opportunity to attest to the viability and benefit of telehealth. Web-engine queries for “telehealth” have increased in the past months, paralleled with the increasing searches for Coronavirus information (Figure-1), these trends cannot be ignored as they are beneficial in promoting a new age of productive healthcare delivery options. At the USC Institute of Urology, we started our telehealth program in 2017. Details of the tele-visit flowchart are reported in Table-1.

**Table 1:** Tele-visit flowchart.

First, patients are selected by symptoms and disease and reason for the clinical consultation (urgent vs routine), A phone call to patients to triage who can be evaluated using the tele-visit tools and who needs a physical evaluation and / or imaging and /or instrumentation. Hardware (laptop, personal computer, smartphone or touchpad), software (specific HIPPA compliant tele-health or teleconference call software) and connectivity for the video visit are required in order to set up the tele-visit appointment. When setting up the tele-visit, the support staff needs to provide all the technical information necessary for a telehealth appointment For new patients, previous health records need to be provided and outside imaging and tests downloaded into the EMR At the time of the telehealth visit an appropriate environment is important to keep the privacy of the patient. At the time of tele-visit, the physician needs to first verify patient's information and obtain verbal consent for the visit. The consent should include purpose and nature of consultation, voluntariness, benefits and risks *(including potential loss of confidentiality)* and potential need for subsequent in-person face to face visit. It is important giving the patient alternatives to tele-visit and notify the patient that other healthcare professionals (including students, residents, and technical personnel) may be involved in the audio-video evaluation. Make sure that patient and physician are online during the entire tele-visit, and fix eventual technical failures in due course. After the visit, the Urologist should document all the information obtained in the medical records. Complete documentation in the medical record of all virtual or phone visits must include: ✓ the reason for the visit ✓ history of present illness ✓ observations/objectives ✓ the assessment and plan for the patient ✓ confirmation of verbal patient consent

### Impact of COVID-19 on Tele-Education

In the COVID-19 era, there has been an increase demand for educational opportunities as clinical volume has slowed. The American Urological Association (AUA) has previously established an online Core Curriculum, which is updated regularly and available to all AUA members. However, new challenges have risen in medical education with the limits in place due to social distancing. Regional and national conferences have been cancelled, including the AUA Annual Meeting. In response, the AUA has published not only the abstracts from the meeting, but also the surgical videos to enhance virtual learning. A webinar program, AUA Live, is being developed as well.

While virtual learning is being increasingly used in post-graduate medical education, it has been a mainstay of many medical school programs for years, especially during the pre-clinical curriculum (28). Many students have preferred the flexibility of recorded lectures in lieu of large in-person lecture halls. However, post-graduate medical education is more traditionally in-person, with clinical experiences, teaching conferences, grand rounds, and morbidity and mortality conferences (29). Now, conferences and presentations have been shifted to virtual formats. Trainees are often included in telehealth visits to maintain their clinical exposure. Surgical residents cannot replace their operative experience, but some are able to augment their learning with simulators (30). Several surveys have been distributed to assess the impact of COVID-19 in urological training, in particular. A questionnaire sent to all Italian urology residents found that on-call activity was not significantly changed, but there were dramatic reductions in outpatient visits and diagnostic procedures for residents at all levels. Senior residents had compromised volumes of surgical procedures (31). A U.S. based survey from the University of Texas, Houston, is pending publication of these results. A survey of urology residents from 58 countries reported that the preferred educational content included guideline updates and surgical videos. The European Society of Residents in Urology published educational alternatives to compromised activities. For example, to temporarily replace surgical activity, the European Association of Urology education section has online courses, surgery videos, and webinars, and the Surgery in Motion School has videos of surgical demonstrations (32).

To address the demand for high-quality education, a number of programs have started online lecture series. Several institutions have publicized their previously internal lectures, while others have created brand new programs. With the increased usage of tele-conferencing applications, inviting speakers from across the country, and even internationally, has become easier to organize and promote. Moreover, these lectures are readily available to those in practice and not solely limited to trainees within academic institutions. A list of publicly available cost-free lecture series is included in Table-2.

**Table 2:** List of available free lecture series.

Institution/Group	Name	Link
Urology institute at University Hospitals/ Case Western Reserve University and SUNY Upstate	Genitourinary Reconstruction Online Learning Series	https://www.uhhospitals.org/medical-education/urology-medical-education/urology-residency/overview/online-learning-series
Educational Multi-institutional Program for instructing REsidents (EMPIRE)	Urology Lecture Series	https://nyaua.com/empire/
Memorial Sloan Kettering Cancer Center	Science Spotlight	https://www.mskcc.org/research/ski/education-training/sciencespotlight
National Cancer institute, Urologic Oncology Branch	Urologic Oncology Grand Rounds	https://twitter.com/NCiCCR_UroOnc
Society of Women in Urology	TeleURO AFRICA 2020	https://swiu.org/swiu-news/teleuro-africa-fpmrs.aspx
University of California, Irvine	Grand Rounds	http://urology.uci.edu/education_grandrounds.shtml
University of California, San Francisco	Urology Collaborative Online Video Didactics (COViD)	https://urologycovid.ucsf.edu/
University of California, San Francisco	Pediatric Urology Fellowship Lectures Online (PedsUroFLO)	https://pedsuroflo.ucsf.edu/
University of Southern California	Urology 60 Minutes	https://www.youtube.com/channel/UCuOf9gTZLObAM7HXHdUSA_Q

## CONCLUSIONS

In the face of unprecedented risks in providing adequate health care to our patients during this current, evolving public health crisis of COVID-19, alternative patient management tools such as telemedicine services, allow clinicians to maintain necessary patient rapport with their healthcare provider when required. As a subspecialty, urology should take full advantage of telehealth and tele-education at this juncture. As tele-urology and tele-education can obviate the potential pitfalls of “social distancing” as it pertains to healthcare, the platform can also reduce the risk of COVID-19 spread, without compromising quality urological care and educational efforts. Telehealth can bring urologists and their patients together, perhaps closer than ever.
